# A time-dependent offset field approach to simulating realistic interactions between beating hearts and surgical devices in virtual interventional radiology

**DOI:** 10.3389/fcvm.2022.1004968

**Published:** 2022-09-23

**Authors:** Haoyu Wang, Jianhuang Wu

**Affiliations:** ^1^Shenzhen Institutes of Advanced Technology, Chinese Academy of Sciences, Shenzhen, China; ^2^Shenzhen College of Advanced Technology, University of Chinese Academy of Sciences, Shenzhen, China

**Keywords:** virtual reality, interventional radiology, heartbeat simulation, angiography, skills training, surgery planning

## Abstract

Endovascular interventional radiology (IR) is a minimally invasive procedure for the treatment of vascular diseases. This procedure requires physicians to be highly skilled at manipulating interventional devices under the guidance of two-dimensional X-ray imaging. By offering a non-error-sensitive and radiation-free environment, a virtual reality-based simulator provides a promising alternative for surgical skills training and surgery planning. Building a realistic and interactive simulator is a challenging task. To achieve better realism, this paper proposes a novel method of simulating the heartbeat for both standard and patient-specific anatomical data. A time-dependent offset field approach is proposed to efficiently and stably simulate the interactive behavior between the dynamic heart mesh and surgical devices. For medical imaging simulation, we propose a GPU-based linear depth subtraction method to approximate fluoroscopic images based on the attenuation of the X-ray. On this basis, a topology-based flow map method is proposed to simulate the propagation of the contrast medium in angiography. Experimental results show that the proposed algorithm can simulate heartbeat stably for meshes with varying geometrical shapes and complexities. In efficiency, the dynamic heart mesh can interact with surgical devices stably at 60 frames/s. Under the simulated fluoroscopic imaging effect, the injected contrast medium can realistically visualize both dynamic and static vessels. In a face validity by medical students and clinicians, the category of effectiveness score 8.35 out of 10 on average, demonstrating that our simulator is useful in surgical skills training and surgery planning.

## Introduction

### Background

In recent decades, endovascular procedures have gained increasing popularity because of their advantages over traditional open surgeries, such as minimum invasiveness, short hospital stays, and fewer complications. However, these procedures require physicians to be highly skilled at manipulating surgical devices under the guidance of two-dimensional X-ray imaging. In addition to achieving a complex understanding of three-dimensional anatomy from two-dimensional displays, physicians must spend years of practice acquiring good hand-eye coordination (1). Traditional training methods (e.g., apprenticeships on patients, using human cadavers or live animals, and employing phantoms) are risky, expensive, and restricted to limited morphological models (2). Moreover, exposure to radiation during these training processes harms trainees’ health.

Interactive virtual reality-based simulators provide a promising solution that addresses all the above difficulties by offering a non-error-sensitive and radiation-free environment in which physicians can practice surgical skills repeatedly (3–5). Research (6) has shown that experiences in simulator training can increase medical students’ enthusiasm for interventional radiology (IR). A recent study (7) demonstrated that patient-specific procedure rehearsal can effectively improve surgical performance and patient outcomes.

### Challenges

Building a realistic interventional simulator for surgical skills training and preoperative rehearsal is a challenging task. Realism, real-time interaction, and the capability of processing patient-specific data are the essential features of such a simulator.

Heartbeat is the vital sign of a live person. Most simulators (8) proposed in recent decades use static polygonal meshes to represent the patient’s circulatory system. The absence of heartbeat results in a lack of realism. However, real-time heartbeat simulation is a challenging task. Most research (9) on this subject focuses on the numerical analysis of hemodynamic and cardiac electrophysiology instead of real-time soft tissue deformation. Therefore, these simulation results cannot be used in interactive applications. Moreover, the heart meshes used in these works are crafted to preserve ventricles, atriums, and valves for complicated mathematical modeling. In contrast, the morphological details of the heart mesh in interventional simulators are limited by the quality of patient-specific computerized tomography (CT) images. Without extra artificial intervention, only the outer surface of the heart and its nearby coronary arteries can be reconstructed. Thus, most of the existing modeling methods cannot be adopted in real-time heartbeat simulation. Handcrafted animations are used in some simulators (10, 11) to achieve higher fidelity, but they are not applicable to patient-specific data. Some work (12) has used 4D (3D + time) CT images to create animated heart meshes. Although better realism is achieved, the access requirement for specific types of images limits this method’s feasibility. As described, realistic and real-time heartbeat simulation for patient-specific data is a challenging but essential task.

The collision detection and response between the dynamic heart mesh and surgical devices is another challenging task (13). Unlike other interactive applications (e.g., video games), the primitive of collision detection in an IR simulator is the triangular facet that composes vascular meshes. On the one hand, the time variation of heart mesh vertices makes collision detection computationally intensive, although many optimization techniques (14) have been proposed to improve the efficiency and accuracy of continuous collision queries. On the other hand, the frequent change in boundary conditions makes it difficult to keep the simulation of the surgical devices stable.

The realism of medical imaging simulation is another criterion used to evaluate an IR simulator. Existing methods (15, 16) use volumetric rendering techniques to simulate X-ray imaging based on three-dimensional image data. Polygonal mesh-based methods (17) that utilize the programmable pipeline of a GPU are also proposed. In these methods, centerlines or hierarchical tree structures are created for vascular meshes to simulate digital subtraction angiography (DSA). With the assumption that all vascular meshes are static, these methods cannot properly handle a dynamic circulatory system involving beating heat meshes.

### Our contributions

In this paper, a simplified heartbeat model is proposed to simulate real-time heartbeat for patient-specific data. The interactive behavior of surgical devices and dynamic heart meshes is simulated stably by the proposed time-dependent offset field approach. A linear depth subtraction algorithm is proposed and implemented in the GPU for the real-time simulation of X-ray imaging. Based on the vascular meshes’ topological adjacency information, a blood flow map is generated to simulate the propagation of the contrast medium under virtual fluoroscopy. Experimental results demonstrate that the heartbeat can be simulated realistically and efficiently for heart models reconstructed from patient-specific data and that the simulated surgical devices interact with the dynamic heart meshes stably in real-time. Face validity by medical students and physicians indicates the feasibility of the proposed simulator in surgical skills training and procedure rehearsal.

Our contributions can be summarized as follows:

1) We propose an efficient and robust heartbeat simulation method for standard and patient-specific anatomical models to achieve higher fidelity in the IR simulator.
2) We propose a time-dependent offset field to simulate the interactive behavior of the dynamic heart mesh and surgical devices.
3) We propose a linear depth-based approach to simulate fluoroscopy in the GPU and a topology-based flow map method to simulate the propagation of contrast medium in DSA.

## Related work

Much work on the development of virtual reality-based simulators has been done since Anderson et al. (18) first proposed their interventional simulator in 1996. Then, Wang et al. (19) proposed a simulator for percutaneous coronary revascularization procedures. They represented the vasculature with a centerline hierarchy model and simulated the behavior of surgical devices with the finite element method (FEM). Dawson et al. (10) also proposed a simulator, called ICTS, for interventional cardiology training. In this simulator, a multibody system is used to represent a catheter and guidewire. A polygonal model associated with the X-ray attenuation coefficient was used to simulate the fluoroscopic visual effect. In the simulator proposed by Cotin et al. (20) an incremental FEM is used to simulate the interaction between devices and the vascular model. An optimization strategy based on substructure decomposition is used to ensure real-time efficiency. In medical imaging simulation, a volume rendering method was proposed to approximate fluoroscopic imaging. Also, Korzeniowski et al. (21) proposed their interventional simulator in which a particle model (22) with adaptive radius was used to simulate the propagation of the contrast medium. Wang et al. (17) proposed a physics-based virtual reality simulator to simulate a guidewire and catheter. An RGB-encoded depth technique was used to approximate the X-ray imaging effect. In addition, they built a hierarchy tree of the vessels with centerline and radius information to simulate the propagation of the radiology contrast medium. Li et al. (23) built a personalized percutaneous coronary intervention simulator. For simulating heartbeat, myocardial fiber orientations are constructed to represent the cardiac dynamic characteristics. In an interventional electrocardiology training system proposed by Talbot et al. (12), 4D heart images were used to create an animated heart mesh, and a bounding volume hierarchy (BVH) proximity approach was proposed to ensure the efficiency and continuity of collision detection.

In addition to simulators proposed by research institutes and universities, there are some commercial interventional simulators (24). Mentice, Surgical Science (formerly Simbonix), and CAE have released commercial interventional simulators: the VIST series (25), ANGIO Mentor (26), and CathLabVR (11), respectively.

Most existing simulators use static polygonal meshes or hierarchical centerlines to represent the vasculature, limiting the realism of the simulation. Although some works (10, 11) use handcrafted animation created with computer-aided design software (Maya, Blender, etc.) to gain higher fidelity, this is not a feasible solution for patient-specific procedure rehearsal. Wu et al. (27) proposed a spatial distortion method to approximate the motion of the heart and the surgical devices inside. However, this method also distorts the heart’s nearby anatomical models, resulting in severe artifacts. The behavior of surgical devices within animated heart meshes is simulated well in (12) based on a BVH that is updated in each simulation step. However, this simulation requires access to the patient’s 4D heart images. Similarly, the heartbeat simulation in (23) also depends on the ECG and US data in addition to CT images. These methods bring extra constraints on the data source.

For medical imaging simulation, the volume rendering-based method proposed by Muniyandi et al. (15) approximates fluoroscopic images by tuning the parameters of the transfer function. Its efficiency drops greatly as the volume size increases. The X-ray simulation method proposed in (17) uses only polygonal meshes to generate essential information for creating fluoroscopic images. However, their DSA simulation algorithm is dependent on hierarchical vascular centerlines, the extraction of which from triangular meshes is another popular research subject (28).

In contrast to Dawson et al. (10) and Talbot et al. (12), the heartbeat simulation method proposed in this paper is built upon a simplified heartbeat model using triangular meshes. Without the requirement of having access to specific types of original images, our method provides better efficiency and flexibility for patient-specific procedural simulation. Instead of constantly updating the BVH (12), the surgical device behavior within the beating heart mesh is simulated with a novel time-dependent offset field method. The reasonable tradeoff between computational resources and memory space ensures the realism, efficiency, and robustness of our simulation. For X-ray imaging, our method is based on the linear depth of the triangular meshes. Realistic fluoroscopic images for both static and dynamic models are generated in real-time. Unlike (17, 21), the proposed angiography simulation method is based on the intrinsic topology information of triangular meshes. Without requirements for a centerline or tree structure representation of the vasculature, our method is more flexible for handling vascular meshes with various morphological features. Experiments and user evaluation results show that our simulator can simulate interventional procedures realistically in real time.

## Methodology

**FIGURE 1:**
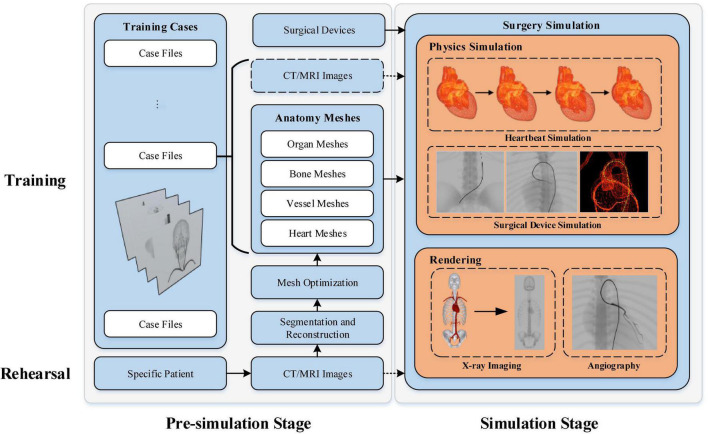
Workflow of our interventional simulator.

As illustrated in Figure 1, the workflow of our simulator has two paths: one for surgical skills training and the other for procedure rehearsal. Each path is divided into two consecutive stages. The anatomical models are prepared at the pre-simulation stage, while the surgical procedures are simulated at the simulation stage.

### Surgical skills training

A collection of clinical cases specialized for training certain types of interventional surgical skills are built. The case files include surgical devices, anatomical models, and optionally patient CT and/or magnetic resonance image (MRI). The simulator imports the digital resources of the selected training case to initiate the simulation. Note that, the CT/MRI images help trainees understand the anatomy’s morphological features for educational purposes. These images are not necessary for the surgery simulation itself.

### Procedure rehearsal

Instead of being imported directly from prebuilt training case files, the anatomical models used for patient-specific procedure rehearsal must be reconstructed from the patient’s CT/MRI image data. The binary masks for the anatomy of interest are first segmented from the input raw images. Then, the segmentation results are used to reconstruct the anatomical triangular meshes. Before being imported for procedure simulation, the reconstructed meshes must be optimized to gain better performance. After selecting the proper surgical devices, physicians can start the patient-specific procedure simulation to rehearse the upcoming surgery.

In this section, we first describe the acquisition of anatomical models for patient-specific data (see section “Anatomical mesh acquisition”). Then, the methods for heartbeat simulation (see section “Heartbeat simulation”), surgical device simulation (see section “Surgical device simulation”), and fluoroscopic simulation (see Section “Fluoroscopy simulation”) are described in sequence.

### Anatomical mesh acquisition

The triangular meshes used in our simulator are generated from patient CT images. The process uses two consecutive steps: image segmentation and 3D reconstruction. As a complex task in medical analysis, medical segmentation takes original CT/MRI images and outputs volumetric binary images for the anatomy of interest. Many traditional and deep learning-based methods of medical image segmentation have been proposed over the last several decades (29). Based on our previous work (30, 31), we use a global context network to produce representative features of the target anatomy and aggregate the contextual information with two attention modules. With the deep learning-based method, the anatomy of interest can be effectively and efficiently segmented.

3D reconstruction takes the volumetric binary images generated in the previous step and creates triangular meshes. Research on 3D reconstruction from medical images has been a popular subject for decades (32, 33). Based on our previous work (34), an adaptive curvature-based method is used to reconstruct the vascular meshes.

Meshes reconstructed from volumetric binary images cannot be directly used for rendering and physics simulation in our simulator. Optimizations including smoothing, simplification, non-manifold edge fixing, and hole filling must be performed before the simulation starts. First, mesh smoothing methods are used to remove the stair artifacts induced by reconstruction algorithms such as the marching cube. Second, the original reconstructed mesh usually consists of an excessive number of facets, which leads to extra computational cost with little improvement in rendering realism and simulation accuracy. Simplification methods are used to reduce the number of facets while preserving the meshes’ intrinsic geometrical details. Third, our DSA simulation algorithm (see Section “Fluoroscopy simulation”) requires the mesh geometry to be a non-manifold to achieve fast and accurate flow map generation. Therefore, all non-manifold edges introduced at the reconstruction stage need to be fixed. Last, our X-ray imaging simulation method assumes that all rendered meshes are closed. Meshes with holes will cause rendering artifacts because their linear depth (thickness) cannot be calculated correctly.

### Heartbeat simulation

**FIGURE 2:**
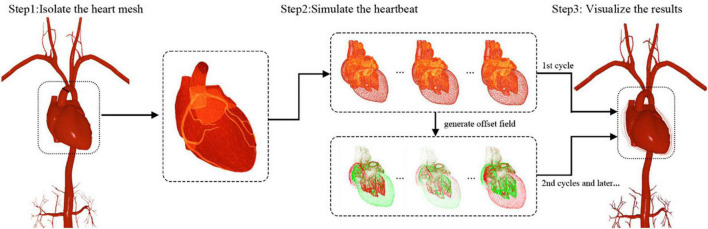
Heartbeat simulation and visualization.

As depicted in Figure 2, the heartbeat is simulated in three steps. Below, we elaborate on all the steps in details.

#### Step 1: Isolate the heart mesh

First, the reconstructed circulatory system is represented by one triangular mesh in which the heart and its biologically connected vessels (the aorta, pulmonary veins, etc.) are also linked topologically. Compared with the magnitude of the heartbeat, the movement of most arterial vessel walls (pulse) is too small to be visually perceived. Therefore, only the heart and its nearest vessel meshes are used for heartbeat simulation, and the rest of the vasculature is considered static in this paper. With this approximation, the mesh for heartbeat simulation is disconnected from the remaining vascular mesh and used as input for the subsequent algorithm, as shown in Figure 2.

#### Step 2: Simulate the heartbeat

The human heart consists of four chambers: two atria and two ventricles. The heart beats because of the contraction and relaxation of the heart muscle. The contraction makes the volume smaller, while the relaxation does the opposite. To approximate this phenomenon, an imaginary point is placed at the geometrical center of each heart chamber. A periodic force in the direction of this point is then exerted on the chamber mesh’s vertices to simulate the volume change of this chamber.

Based on the work of Wang et al. (35), we use a simplified model of the heartbeat, two piecewise trigonometric functions, to describe the contraction and relaxation of the atrium and ventricle. A cardiac cycle is divided into eight deformation stages. For the atrium, the function is defined as:


$$\varphi_{\mathrm{Atrium}}=\left\{ \begin{array}{c} \text{cos}\left(\pi \cdot \text{t}\right),t\in \left[0,1\right)\\ \text{cos}\left(\pi \cdot \text{t}/7+\frac{6}{7}\pi \right),t\in \left[1,8\right) \end{array}\right.$$

For the ventricle, the function is defined as:


$$\varphi_{\mathrm{Ventricle}}=\left\{ \begin{array}{c} \text{cos}\left(\pi \cdot \text{t}/5+\frac{44}{25}\pi \right),t\in \left[0,1\right)\\ \text{cos}\left(\pi \cdot \text{t}/3-\frac{2}{5}\pi \right),t\in \left[1,4\right)\\ \text{cos}\left(\pi \cdot \text{t}/5+\frac{4}{25}\pi \right),t\in \left[4,8\right) \end{array}\right.$$

The force exerted on the vertex of the chamber with index i is defined as:


$${\text{F}}_{i}=\text{k}\left(\left|{\text{x}}_{c}-{\text{x}}_{i}\right|\right)\cdot \frac{{\text{x}}_{c}-{\text{x}}_{i}}{\left|{\text{x}}_{c}-{\text{x}}_{i}\right|}\cdot \varphi$$

where **x_i_** is the vertex position, **x_c_** is the geometric center of the chamber, and *k* is the elastic coefficient. To obtain a smooth surface during deformation, we add two stretching constraints: the distance between adjacent vertices and the distance from a vertex’s current position to its initial position (36). Additionally, volume conservation constraints are added to preserve the heart volume during deformation. Within the framework of position-based dynamics (PBD) (37), the stable shapes of the heart for a complete cardiac cycle can be calculated iteratively.

The experimental results (see section “Heartbeat simulation”) demonstrate that the heartbeat can be simulated in real time for meshes within a limited number of vertices. Nevertheless, the simulation is computationally intensive, and its efficiency drops as the number of vertices increases. As another key component of a realistic IR simulator, the simulation of surgical devices also requires numerous computations. The finite CPU resources must be distributed properly to ensure the real-time efficiency of the whole simulation. The deformation of the heart repeats itself periodically. If the parameters of the simulation remain the same, it is not necessary to repeatedly calculate the shapes of the heart except in the first cardiac cycle. To achieve higher efficiency and reserve enough computational resources for the surgical device simulation, a time-dependent offset field is created to cache the simulation results of a complete cardiac cycle for rendering and subsequent simulation.

**FIGURE 3:**
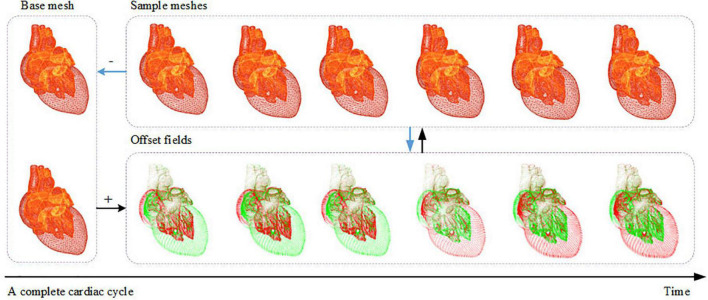
Generation of the time-dependent offset field.

As illustrated in Figure 3, a list of heart meshes (*S*_1_, *S*_2_, …, *S*_*M*_) is sampled from the simulation results for the first complete cardiac cycle. The first sample *S*_*1*_ is selected as the base mesh, whose vertex position is treated as the baseline for offset. For the *i*th vertex ${\text{v}}_{i}^{j}$ of the *j*th sample mesh, the positional offset $\sigma_{i}^{j}$ from its counterpart ${\text{v}}_{i}^{1}$ in the baseline mesh can be calculated as $\sigma_{i}^{j}={\text{v}}_{i}^{j}-{\text{v}}_{i}^{1}$. The offset vectors are depicted with short colored lines in Figure 3. The vector in red is in the same direction as the normal of the vertex, indicating that the chamber’s volume increases due to heart muscle relaxation. Conversely, the vector in green points in the opposite direction of the vertex normal, indicating a decrease in the chamber’s volume caused by the contraction of the heart muscles. The periodic change of the heart chamber’s volume results in a realistic heartbeat simulation. For a cardiac cycle, the direction and magnitude of the offset vectors change over time due to the deformation of the heart mesh, forming a time-dependent offset field in 3D space.

#### Step 3: Visualize the results

As depicted in Figure 2, the simulation results are visualized with different inputs for the first and subsequent cardiac cycles. In the first cycle, the deformed heart meshes are calculated and used to generate the time-dependent offset field. These meshes are uploaded to the GPU for visualization. Starting in the second cycle, the instantaneous offset vector for a vertex of the heart mesh can be fetched from the newly generated offset field with the vertex index *i* and a normalized time parameter *t* ∈ (0, 1). By adding this offset vector to the position for every vertex of the base mesh, we can calculate the shape of the deformed heart at any instant of a cardiac cycle at a low computational cost. Moreover, the calculation can be further optimized by uploading the offset field to the GPU and adding offset vectors in parallel for each vertex.

As mentioned above, the offset field remains invariant unless the parameters of the heartbeat simulation change. Hence, for a cardiac cycle, the heart meshes are calculated once, and the results are cached by creating a time-dependent offset field. The subsequent heartbeat simulation and visualization run completely in the GPU, reserving sufficient computational resources in the CPU for surgical device simulation.

With this method, the heartbeat for any patient-specific data can be simulated realistically and efficiently. Note that, the magnitude and rate of the heartbeat can be changed by tuning the parameters to simulate the human body’s reaction to stimulation in surgical procedures.

### Surgical device simulation

One of the most important tasks in building an IR simulator is simulating the interactive behavior between the circulatory system and surgical devices. As explained in section “Anatomical mesh acquisition,” we represent the circulatory system with triangular meshes that are suitable for rendering and collision detection. For different types of interventional devices, which in most cases are long, thin, and hollow, we model them as rigid rods linked by points (**p_0_**, **p_1_**, **p_2_**, …, **p_n_**) that carry the discretized material properties (38). Based on Hook’s law, we formulate the total energy of the device-circulatory system. By iteratively minimizing the total energy, we calculate the devices’ equilibrium shape, which is represented as a list of 3D positions (**x_0_**, **x_1_**, **x_2_**, …, **x**_*n*_).

Instead of constantly performing collision detection, which is very time-consuming for an algorithm with *O*(*n*^3^) time complexity such as ours, we propose a deviation-feedback approach to update the external forces exerted on the device centerline points as in (39). With an adaptive feedback coefficient based on the centerline points’ historical displacements, the simulation converges within fewer iterations, and the simulated device behaves stably in vascular models with complex morphological features.

In endovascular procedures, multiple interventional devices (guidewire, catheter, etc.) are usually manipulated in turn to reach certain places in the circulatory system. To represent nested interventional devices, we propose a shared-centerline model in which the centerline points are shared while their material properties are blended. With this model, the interaction between nested interventional devices is simulated by dynamically blending the material properties of the shared centerline points.

With the incorporation of the heartbeat, the circulatory system now comprises static vascular meshes and a dynamic heart mesh. When they are advanced into the heart area, the simulated interventional devices must interact with the dynamic mesh realistically and stably in real time. The shape of an interventional device is maintained when it collides with vessel walls. It is observed that the devices tend to move along with periodically moving vessel walls instead of bouncing back and forth inside the vascular tunnels of the beating heart mesh. Utilizing the time-dependent offset field, we propose a visual-deviation method to simulate the synchronous movements of surgical devices and the beating heart. The method is explained in Algorithm 1.

**Algorithm 1:** Simulation of the interaction between surgical devices and beating heart meshes.

**Simulation stage:** For each point ***p_i_*** in device centerline *D_p_*: Perform collision detection between ***p_i_*** and the baseline mesh *S*_1_ to obtain the spatial neighbor vertex index collection *C_i_* of ***p_i_***. Calculate the stable shapes of the devices (***x*****_0_**, ***x*****_1_**, ***x*****_2_**, …, ***x***_*n*_). **Visualization stage:** Obtain the normalized time parameter *t* of the heartbeat. For each point ***p_i_*** in the device centerline: For each vertex index *j* in *C_i_*: Obtain the offset vector ***σ_j_(t)*** for vertex ***v*****_j_** of mesh S_1_ from the offset field at time *t*. Calculate the visual offset $\delta_{i}\left(t\right)=\frac{1}{n}\sum_{j=1}^{n}\sigma_{j}\;(t)$. Calculate the new device centerline *D_r_* by adding (***δ***_**0**_, ***δ***_**1**_, ***δ***_**2**_, …, ***δ***_***n***_) to (***x*****_0_**, ***x*****_1_**, ***x*****_2_**, …, ***x***_*n*_). Render *D_r_*.

**FIGURE 4:**
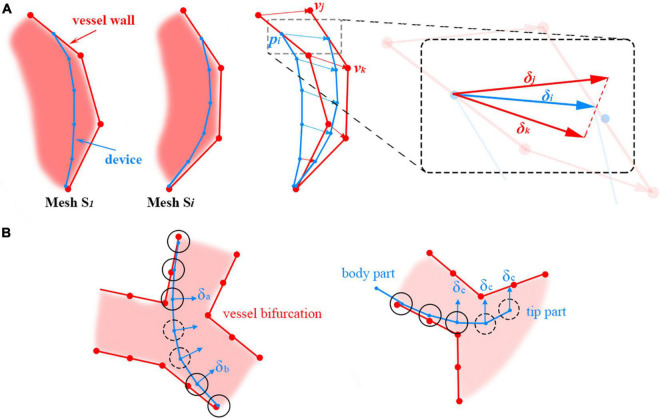
**(A)** Adding positional deviations to the device centerline to make it move along with the beating heart. **(B)** Offset vector calculation for centerline points (floating points) without the surrounding heart mesh vertices. The floating and non-floating points of the centerline are circled with dotted and solid lines, respectively.

When the simulation starts, the baseline mesh in the heartbeat simulation is used to perform collision detection and calculate the stable shape of the device centerline. Meanwhile, we can obtain the neighboring vertices of the heart mesh for each point **p_i_** of the centerline. Thus far, the calculated device centerline is static. To make the devices move synchronously along with the beating heart, a time-dependent offset vector is added to every point of the centerline based on the progress of the heartbeat, as shown in Figure 4A.

For example, **x_i_** is the stable position of point **p_i_** on the centerline. There are *n* vertices of the mesh *S*_1_ in the spatial neighborhood of **p_i_**, and their indices are *C*_*i*_ = {*c*_1_, *c*_2_*c*_*n*_}. The instantaneous offset vector **σ_j_**(*t*) for the heart mesh vertex with index *j* ∈ *C*_*i*_ at time *t* can be retrieved directly from the time-dependent offset field generated in the heartbeat simulation. The average offset vector for the vertices in *C_i_* is calculated and then added to **x_i_** to visually deviate the centerline to synchronize the movement of the devices and the heartbeat.

In the simulation, there are centerline points that are not directly in collision with vessel walls. Thus, no heart mesh vertices can be found in their close neighborhoods, and their offset vectors cannot be directly calculated from the offset field. For brevity, these points are referred to as ‘floating’ points. Floating points are usually located on a sharp curve at vessel bifurcations and at the tip of the device; they are circled with dotted lines in Figure 4B left and right, respectively. Different methods are used to calculate the offset vectors of these floating points under these two circumstances. For floating points in the middle of the device centerline, the offset vectors are calculated by interpolating their adjacent non-floating points’ offset vectors δ_**a**_ and δ_**b**_, as shown in Figure 4B left. Smooth transitions between the line segments separated by the floating points can be obtained in this way. For floating points at the tip of the device centerline, the offset vector δ_**c**_ for the closest non-floating point is used directly, as shown in Figure 4B right.

In summary, the baseline mesh for heartbeat simulation is used for collision detection and surgical device centerline calculation. Utilizing the time-dependent offset field, the surgical device centerline is visually deviated to synchronously move with the beating heart, resulting in realistic, efficient, and stable simulation results for the interactive behavior between surgical devices and the dynamic heart mesh.

### Fluoroscopy simulation

As an important part of IR procedures, fluoroscopy is a continuous X-ray imaging technique that shows the movement of a body part (e.g., heart) or the course that a medical instrument or dye (contrast medium) takes as it travels through the body (40). In this paper, we propose a geometry-based method to simulate X-ray imaging. On this basis, we propose a topology-based flow map method to simulate the propagation of the contrast medium under the fluoroscope.

#### X-ray imaging simulation

Based on the physics of X-ray imaging (41), an X-ray image shows the variations in transmission caused by structures in an object of varying thickness, density, or atomic composition. To approximate different anatomies’ attenuation in X-ray imaging, we introduce a weighted thickness variable μ, which is defined as μ = *sd*, where *s* and *d* are the weight and linear thickness of the anatomy, respectively.

The proposed X-ray imaging simulation method takes two render passes: one for creating the weighted thickness texture for all anatomical models and the other for creating fluoroscopic images.

#### Pass 1: Create the thickness texture

In this pass, the linear thickness *d* of the rendered anatomy model is first calculated for each pixel by subtracting its front faces’ linear depth from that of its back faces. The formula is defined as *d* = *z*_*back*_−*z*_*front*_, where *z_front_* and *z_back_* are the z components of the pixel’s camera space position for the front and back faces, respectively. Then, the thickness is multiplied by the anatomy-specific weight value *s*. The weighted thicknesses for all rendered anatomies are summed, and the results are written to a rendered texture (target) in float format. In this way, the sum of the weighted thickness for any pixel can be read directly from the texture.

#### Pass 2: Create the fluoroscopic image

In this pass, the grayscale *g* for each pixel is calculated based on the attenuation formula, defined as


$$\text{g}={\text{e}}^{-a\sum \mu }$$

where *a* is the uniform attenuation factor and ∑μ is the summed weighted thickness obtained from the texture created in the first render pass. By selecting proper values of the weight *s* for different anatomies, X-ray imaging can be simulated realistically at a very low cost.

Note that, the depth value in the camera space is used for the thickness calculation because the normalized depth value retrieved directly from the Z-buffer of the rendering pipeline is non-linear and suffers from precision problems. Because both the front and back faces are needed to calculate the thickness, all triangular meshes must be closed. Otherwise, there will be artifacts in the holes of the unclosed meshes.

Unlike existing image-based methods, the proposed algorithm for X-ray imaging simulation takes only triangular meshes as input and runs in parallel at the pixel stage of the rendering pipeline, ensuring real-time efficiency. Moreover, the vertices of the heart mesh are offset for heartbeat rendering at the vertex stage, the output of which can be used directly as input for the pixel stage to simulate X-ray imaging. The coherency of the pipeline means that our method works for both static and dynamic meshes.

#### Digital subtraction angiography simulation

Digital subtraction angiography is a fluoroscopic technique used extensively in IR for visualizing blood vessels. Contrast medium is injected into the vasculature through catheters, and the affected blood vessels are visually enhanced in dark gray. DSA is one of the most commonly used techniques in IR, and it is necessary to realistically simulate this procedure for building a simulator with high fidelity.

The contrast medium propagates downstream with the blood flow from the tip of the angiography catheter. Only the vasculature that the contrast medium flows through can be visualized. In this paper, a gradient-based region growing method is proposed to simulate this process with triangular meshes. The idea is to find the submesh that is affected by the contrast medium and render it progressively under the fluoroscope to approximate the propagation of the contrast medium. This method can be divided into three steps.

#### Step 1: Build the flow map of the arterial blood

To create the downstream vascular mesh visualized by the contrast medium, the direction of the blood flow must be calculated everywhere in the circulatory system. In this paper, an attribute called a ‘sequence’ is introduced for the triangular facets. The gradient of the sequence approximates the direction of the blood flow nearby. After the sequences for all facets are calculated, a blood flow map can be built for the triangular mesh.

**FIGURE 5:**
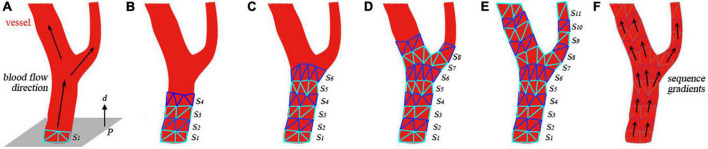
Generation of the submesh of the contrast-medium-affected vascular mesh. **(A)** Initial settings of the calculation. **(B–E)** Intermediate results of the calculation. **(F)** Visualization of the sequence gradients.

As shown in Figure 5, a seed plane P is defined at the root of a vessel with two branches. The plane’s normal **d** points in the same direction as the blood flow. By performing collision detection between P and the vascular mesh, we can obtain a looped triangle strip that intersects with the plane. Let us define this strip as *S*_1_ = {*f*_0_, *f*_1_, …, *f*_*r*_}, where *f_i_* is the triangle’s index in the vascular mesh. In the direction of **d**, we can obtain $S_{1}^{\prime }$s adjacent strip *S*_*2*_, defined as {*f*_*r* + 1_, *f*_*r* + 2_, …, *f*_*s*_}. Based on the breadth-first search (BFS) algorithm, we can obtain a list of strips {*S*_1_, *S*_2_*S*_*N*_}, as shown in Figures 5A–E. For clarity, the adjacent layers of the strips are alternately colored cyan and blue. The strip’s index is used as the value of the sequence for each facet of this strip. As shown in Figure 5F, the direction of the sequence’s gradients is in accordance with that of the blood flow.

#### Step 2: Generate the contrast-enhanced vascular submesh

The contrast medium is injected into the vasculature and propagates with the blood flow to visualize the vessels downstream under fluoroscopy. After building the flow map of the blood in the first step, a sequence value is assigned to every facet of the vascular mesh. Let us define **x_b_** as the first point of the catheter centerline’s body part (distinguished from the intrinsically curved tip part). The closest facet of the vascular mesh to the point **x_b_** is *f_b_*. The direction of the sequence gradient of facet *f_b_* is defined as **n_b_**. Starting from the seed plane defined by **x_b_** and **n_b_**, a list of triangle strips is collected by the region growing in the direction of the sequence gradient. By merging all these strips, we obtain an ordered collection of facets that defines the contrast-medium-affected submesh of the vascular model. The order of the facets approximates the propagation of the contrast medium.

#### Step 3: Render the submesh progressively with a time-varying thickness weight

**FIGURE 6:**
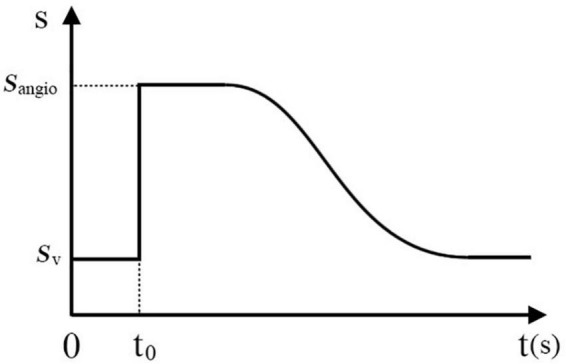
Variation in the weight value over time when the contrast medium is injected.

At the rendering stage, the sorted triangles are uploaded to the GPU and rendered incrementally over time. The number of rendered facets over time n(t) is defined as follows:


$$\text{n}\left(t\right)=\text{v}\left(\text{t}-{\text{t}}_{0}\right)$$

where *t*_0_ is defined as the moment when the contrast medium is injected and ν is the number of facets rendered per second. To simulate the contrast medium’s influence on X-rays, we use a higher value *s*_*angio*_ to replace the vessel’s initial weight *s_v_* for thickness calculation when the submesh is being rendered. As shown in Figure 6, the weight value jumps from *s_v_* to *s*_*angio*_ at *t*_0_. The weight value decreases over time to simulate the dilution of contrast medium in the blood.

## Experiments and discussion

### Experimental setup

The experiments described in this section were conducted in our homemade IR simulator which are composed of haptic feedback devices and a consumer-level laptop (CPU: Intel Core i5, RAM: 16 GB, GPU:NVIDIA GeForce MX450).

The vascular models are reconstructed from real patients’ CT images. The interventional devices are the most commonly used types in daily interventional procedures. The intrinsic material properties of the devices are selected by trial and error offline. One unit for the vascular model in the coordinate system of the virtual world represents one millimeter in the real world.

### Heartbeat simulation

**FIGURE 7:**
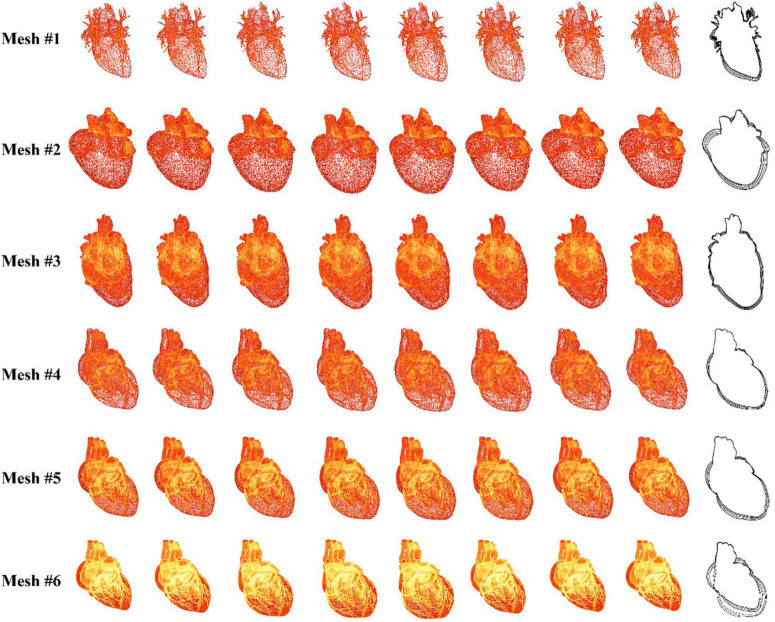
Simulation results for six sets of data.

In this experiment, six different heart meshes are used as input to run the proposed heartbeat simulation algorithm. Meshes 1–4 are reconstructed from four different patients, while meshes 5 and 6 originate from the same patient but with different degrees of simplification (Table 1). As shown in Figure 7, eight samples are taken in the first cycle of the simulated heartbeat to generate the time-dependent offset fields for each heart mesh. To demonstrate the magnitudes of the simulated heartbeats and the geometrical variation between adjacent samples, we merge the silhouettes of the samples into one image, which is placed at the end of each row in Figure 7. Additionally, we record the frame rates of the simulation for the first and the later cycles of the heartbeat to validate the efficiency of our algorithm. Different elastic coefficients (see Section “Surgical device simulation”) are used for meshes 4–6 to change the magnitude of the heartbeat to demonstrate the flexibility of our algorithm.

**TABLE 1:** Configuration and results for input heart meshes.

Mesh	Vertex count	Face count	Elastic coefficient	1st cycle fps	2nd and later fps
1	27369	54566	1.0	35	60
2	29438	58880	1.0	32	60
3	81145	162498	1.0	24	60
4	44452	88727	1.0	28	60
5	177622	354876	1.5	10	60
6	710151	1419576	2.0	2	47

Figure 7 shows that there are no broken or flying vertices in the samples for any of the six meshes, indicating that our algorithm is stable for meshes with varying geometrical shapes and complexities. The shape variation between adjacent samples increases from mesh 4 to 6, showing that we can simulate heartbeats with larger magnitudes by using larger values of the elastic coefficient. To simulate the heart rate change induced by stimulation, we can simply modify the rendering parameters instead of recalculating the heart meshes for a complete cycle (for detailed information, see the Supplementary Video).

With regard to efficiency, our algorithm can simulate the heartbeat at an interactive rate for meshes 1–4. The frame rate drops dramatically as the number of vertices increases from mesh 4 to 6. This is because the increase in the vertex number greatly slows the proposed algorithm by introducing additional constraints and the resulting computation. However, once the time-dependent offset field is built in the first cycle, the frame rate jumps to 60 frames per second (fps) for meshes 1–5. Even for mesh 6, the simulation achieves interactive rates. Because the heart mesh vertices are offset in parallel in the GPU, this method is less sensitive to a change in the number of vertices within a certain range. With this method, many of the computational resources of the CPU are saved for simulating the behavior of surgical devices, ensuring that the surgical procedure can be simulated in real time.

In summary, our method can simulate the heartbeat stably and realistically. By tuning the parameters, we can change the heart rate and the magnitude of the heartbeat. The incorporation of the simulated heartbeat builds a dynamic circulatory system, achieving higher realism for our simulator with little CPU cost.

### Device simulation

**FIGURE 8:**
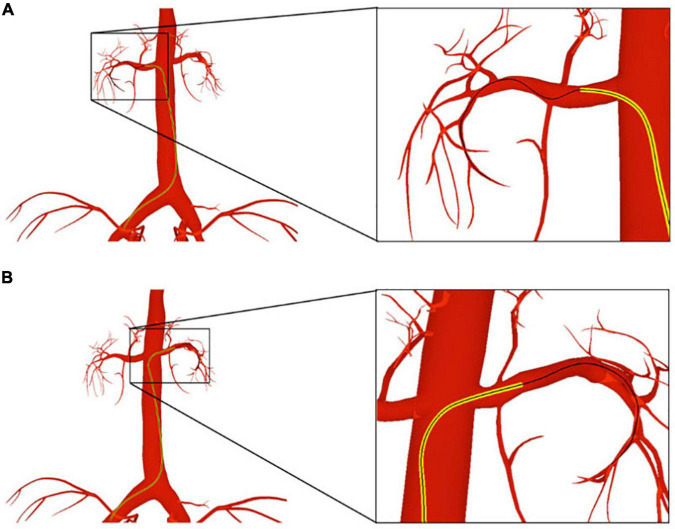
The catheter and guidewire are navigated from the femoral artery to the left **(A)** and right **(B)** renal artery branches. The catheter is displayed as a yellow tube, while the guidewire is represented as a black wire.

The femoral artery is one of the most commonly used access points for physicians to start endovascular procedures. In this experiment, we navigate a catheter and guidewire from the femoral artery to three clinically typical target vessels to validate our device simulation algorithm. As shown in Figure 8, with proper manipulation, the guidewire is advanced from the femoral artery to the left and right renal artery branches. The results show that the simulated devices behave stably when advanced from trunk vessels with large diameters to subsequent narrow branches. Our simulation algorithm is robust enough to handle complex morphological situations (for detailed information, see the Supplementary Video).

**FIGURE 9:**
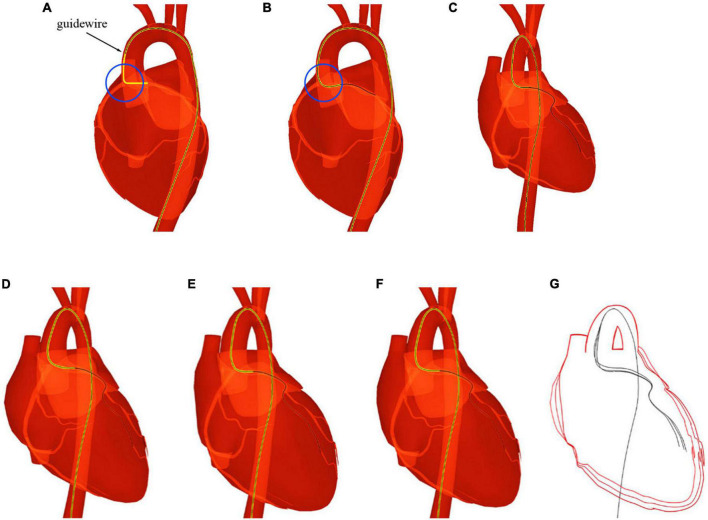
The catheter and guidewire reach the left coronary artery from the femoral artery. **(A)** Before the guidewire passes through the intrinsic curved part of the catheter. **(B)** After the guidewire passes through the intrinsic curved part of the catheter. **(C)** The guidewire reaches the terminal branch of the left coronary artery. **(D–F)** Screenshots taken at different moments of a cardiac cycle. **(G)** Shape variation of the heart and devices.

As shown in Figure 9, we advance the devices to the left coronary artery, which is another important area in clinical practice. The catheter and guidewire must be manipulated in coordination to enter the left coronary artery. By comparing Figures 9A,B, we can see that the guidewire straightens the catheter’s intrinsic curved part (circled in blue) while the guidewire is advanced out of the catheter. This indicates that the existence of the guidewire makes the weighted property of the shared centerline more resistant to bending than the catheter itself. This validates the effectiveness of our shared centerline model. From another perspective, Figure 9C shows the devices’ final shape when the guidewire reaches the target branch vessel, the diameter of which is ten times smaller than that of the aortic arch. The robustness of our algorithm is further proven.

When navigated into the cardiac region, the simulated devices move along with the beating heart in synchronization. Three samples are taken at different moments of a cardiac cycle, as shown in Figures 9D–F. To make the shape variation of the heart and devices more evident, we display the devices’ centerlines and the hearts’ silhouettes in Figure 9G. The device shape remains stable, and the relative distances to the colliding vessel walls remain visually unchanged. In addition, the frame rate remains at 60 fps while the simulated devices are interacting with the beating heart. The results demonstrate that our method can simulate the behavior of interventional devices realistically and stably under dynamic and static mesh circumstances in real time.

### Fluoroscopy simulation

#### X-ray imaging simulation

**FIGURE 10:**
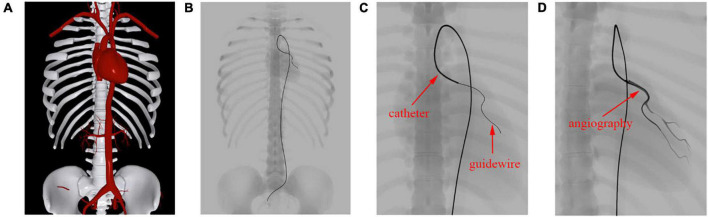
Simulation results of fluoroscope imaging. **(A)** Anatomy rendered in the Phong lighting model. **(B)** Anatomy and surgical devices under a simulated fluoroscope. **(C)** Zoomed view of the devices in the left coronary artery. **(D)** Contrast-enhanced coronary artery vessels.

In this experiment, a cardiac training case is imported into our simulator. The imported meshes contain a skeleton, vessel, and heart. The devices used in this procedure are a catheter and guidewire. The anatomy rendered in the Phong lighting model is displayed in Figure 10A. The corresponding simulated fluoroscopic image is shown in Figure 10B. We move the virtual camera closer to the devices and take a screenshot, which is shown in Figure 10C. Then, angiography is simulated, and the enhanced coronary artery vessels are displayed in Figure 10D.

The skeleton, vessels, and heart mesh can be clearly seen in Figure 10A, while only the skeleton can be identified in Figure 10B. This is because the skeleton’s attenuation in X-ray is much higher than that of the vessels. Unlike human anatomy, surgical devices can be easily found under the fluoroscope because of their specialized materials. The catheter is a hollow tube device with a larger diameter than that of the guidewire, and it can be treated as solid. Thus, we can distinguish these devices by the width of the centerline in Figure 10C. After contrast medium is injected through the catheter, the affected vessel branches are visualized in a dark color under the fluoroscope, as shown in Figure 10D. The frame rate remains at 60 fps regardless of whether the fluoroscope effect is enabled. These results demonstrate that our algorithm can efficiently simulate the X-ray imaging effect of objects with varying properties by tuning the weight value *s* as explained in section “Fluoroscopy Simulation.”

#### The coronary angiography simulation

In this experiment, we simulate the coronary angiography procedure. First, we create the flow map for a complex vascular system that includes the aorta, the carotid artery, the renal artery, and the femoral artery. By placing the seed plane at the beginning of the aorta, we calculate the value of the sequence for all triangles that compose the arterial vessels.

**FIGURE 11:**
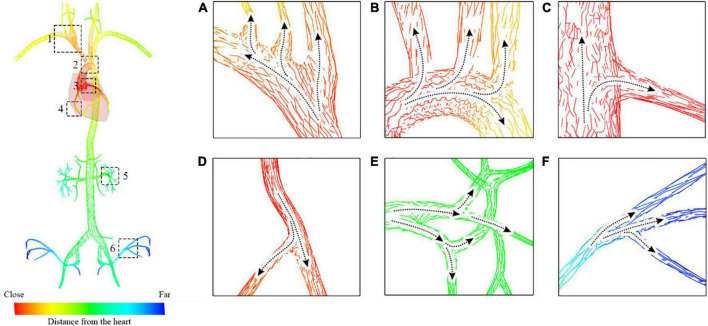
Blood flow map starting from the aorta to the carotid artery and the femoral artery. **(A)** Subclavian artery blood flow. **(B)** Aortic arch blood flow. **(C,D)** Coronary artery blood flow. **(E)** Renal artery blood. **(F)** Iliac artery flow.

As shown in Figure 11, the gradients of the sequence are depicted with short lines colored from red to blue, indicating the facet’s topological distance to the heart from close to far as well as the blood flow from the aorta to the branch vessels. In Figures 11A–F, there are lines pointing from the trunk to the branches at vessel bifurcations with various morphological features. This demonstrates that the gradient can help solve the propagation problem at the bifurcation of blood vessels without building hierarchical tree structures or extracting vascular centerlines.

**FIGURE 12:**
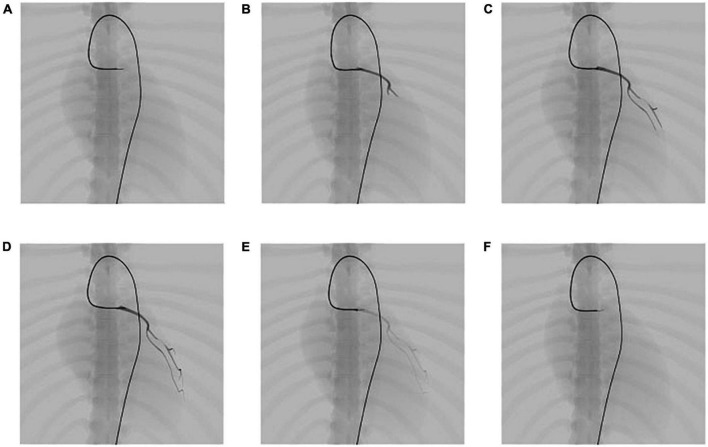
Simulation results of the propagation of contrast medium under fluoroscopy. **(A)** Angiography catheter reaches the entrance of the left coronary artery. **(B–D)** Propagation of the contrast medium and corresponding fluoroscopic images. **(E,F)** Dilution of the contrast medium and corresponding fluoroscopic images.

The catheter is then navigated to the entrance of the left coronary artery, and the contrast medium is injected through the catheter to visualize the coronary vessels. The screenshots shown in Figure 12 are taken at different stages of the procedure, showing the life cycle of the contrast medium from injection to dilution (for detailed information, see the Supplementary Video).

The propagation of the contrast medium is progressive. It runs from the injection point to its downstream vessel branch in the direction of blood flow and dilutes in the blood over time. After the angiography catheter reaches the entrance of the left coronary artery, the contrast medium is injected through the catheter, as shown in Figure 12A. Then, a submesh of the coronary artery is generated progressively and rendered using our fluoroscopy simulation algorithm with a high weight value *s*_*angio*_, as explained in section “Fluoroscopy simulation.” Figures 12B–D) shows the simulation result of the contrast medium’s propagation. As time passes, the contrast medium starts to dilute, and the color of the affected submesh begins to fade, as shown in Figures 12D–F). Note that, the coronary artery is also dynamic because it is a part of the heart.

### Angioplasty simulation

**FIGURE 13:**
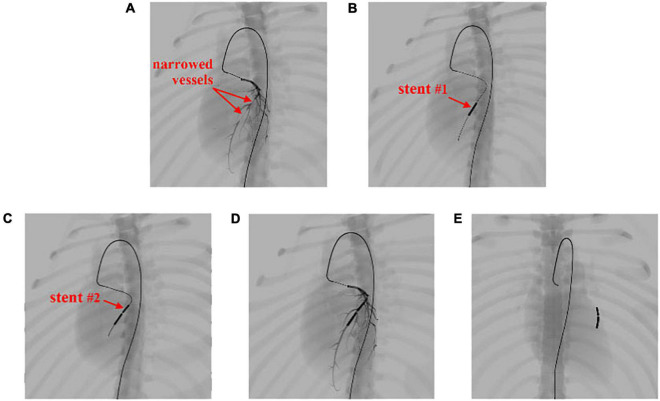
Simulation of the coronary artery stent implantation procedure. **(A)** Visualization of the narrowed vessels with simulated angiography. **(B,C)** Results of stent implantation for the two narrowed vessels. **(D)** Visualization of the post-operative results with simulated angiography. **(E)** The implanted stents stay within the coronary artery and move with the beating heart.

In this experiment, we perform coronary angioplasty and stent placement for the heart. There are two blockages in the patient’s left coronary artery. In this procedure, we navigate the catheter and guidewire into the left coronary artery and implant two stents to open the narrowed vessels. Screenshots of key moments of the procedure are shown in Figure 13.

In Figure 13A, the surgical devices reach the entrance of the left coronary artery. After the injection of the contrast medium, we can see two areas in which plaque blocks the blood flow of the visualized vessels. Balloon catheters are then advanced to the narrowed vessel to implant two stents consecutively from the far end to the near end of the vessel branch, as shown in Figures 13B,C. Contrast medium is injected again to visualize the coronary artery for observation of blood flow after the procedure, as shown in Figure 13D. Then, the surgical devices are pulled out of the coronary artery. The stents stay where they are placed and move along with the pumping heart, as shown in Figure 13E. The frame rate stays at 60 fps for the whole simulation time. The results demonstrate that our simulator can simulate complex endovascular procedures realistically in a dynamic circulatory system with real-time efficiency (for detailed information, see the Supplementary Video).

### Face validity

To validate the effectiveness of our simulator, 24 participants with varying degrees of expertise in IR are recruited to evaluate our simulator. A questionnaire is designed for participants to grade our simulator in six respects: the virtual anatomical environment, device behavior, visualization, efficiency, robustness, and effectiveness. The criteria for the scores are as follows: 0–2 means strongly disagree; 3–5 means disagree; 6–8 means agree, and 9–10 means strongly agree. The average score in each category is listed in the second column of Table 2.

**TABLE 2:** Numerical metrics of the user evaluation.

Category	Score
(1) Virtual anatomical environment	
(1.1) The heartbeat is realistic.	8.3
(1.2) The reconstructed vessels are accurate.	9.6
(1.3) The reconstructed skeleton is accurate.	9.7
(2) Device behavior	
(2.1) Devices’ interaction with vessel wall is realistic.	9.0
(2.2) Interaction between devices is realistic.	9.1
(2.3) Devices’ interaction with the beating heart is realistic.	8.6
(2.4) Devices’ behavior at vessel bifurcation is realistic.	8.9
(2.5) Devices’ behavior at narrow branches is realistic.	9.6
(3) Visualization	
(3.1) Fluoroscopy effect is realistic.	8.7
(3.1.1) Anatomy under fluoroscope is realistic.	8.3
(3.1.2) Devices under fluoroscope are realistic.	9.4
(3.2) Propagation of contrast medium is realistic.	8.6
(4) Efficiency	
The simulation runs smoothly without lag.	9.8
(5) Robustness	
The simulation runs stably without crash or exception.	9.7
(6) Effectiveness	
(6.1) The simulator is useful for surgical skills training.	8.5
(6.2) The simulator is useful for surgery planning.	8.2

In the first category, approximately 80% of the participants agree that the simulated heartbeat is realistic and its incorporation greatly increases the realism of our simulator. The other 20% of participants, who have more than 10 years of clinical experience, suggest that the heartbeat should not only be visually plausible but also be numerically accurate for further application in hydrodynamic analysis. Almost all participants give a high rating to the accuracy of our reconstructed vessels and skeletons after a thorough cross-reference with the original CT images.

The device behavior in our simulator obtains a high rating, especially in the interaction with vessel walls, bifurcation, and narrow branches. The average score of the device behavior inside the beating heart is not as high as the other scores because experienced participants expect to see how the devices interact with the blood flow pumped out of the heart. However, our simulator does not incorporate real-time blood flow simulation, which is another very challenging task. Nevertheless, good ratings are received in the device behavior category.

In the category of fluoroscopy simulation, more than 90% of the participants agreed that the results are realistic enough for training and surgery planning purposes. Approximately 5% of the participants suggest that more anatomical parts should be involved in visualization to enrich the simulated fluoroscopic images even if they might not be visually evident as skeletons and vessels affected by the contrast medium.

All participants agree that our simulator runs quickly and that no operational lag is detected in the whole procedure. In addition, no vital errors that can corrupt the simulation are found. The numerical metrics in Table 2 show that participants reached a consensus in terms of the stability of our simulator.

In summary, participants with less clinical experience give our simulator high rankings on average. They believe that our simulator is a very useful tool for training interventional skills because it is realistic, reusable, and free of radiation and ethical risks. In comparison, participants with more experience are more interested in the surgery planning capability of our simulator and set a much higher standard for us. Nevertheless, they agree that our simulator’s ability to process and import patient-specific data for simulation can offer much help in making surgery plans.

## Conclusion

In this paper, we propose a novel approach to simulating the heartbeat for building a realistic and interactive IR simulator. A simplified heartbeat model is used to formulate the driving force of the heartbeat. To achieve better efficiency, a time-dependent offset field is built to cache and reproduce the simulation results. The interaction between surgical devices and the beating heart is simulated realistically and stably with the proposed visual-deviation method. For X-ray imaging, a linear depth subtraction method is proposed to approximate the fluoroscopic images with an anatomy-specific weight value. For angiography simulation, a blood flow map and a gradient-based region growing method are used to generate the contrast-enhanced vascular meshes. A progressive rendering technique with a time-varying weight value is used to render these meshes for simulating the propagation of the contrast medium. To summarize, the proposed methods solve three challenging simulation problems which are the real-time heartbeat simulation for patient-specific data, the stable interaction between dynamical meshes and surgical devices, and the realistic medical visualization and angiography. Experimental results demonstrate that our approach is capable of realistically simulating the heartbeat and its interaction with surgical devices in real time. The simulation results of the angioplasty for the left coronary artery validate the realism and efficiency of the proposed methods for X-ray imaging and angiography simulation.

Despite the increase in the realism of our simulator, there are still some problems to solve for achieving better fidelity and functionality. Blood flow simulation is one of the most essential but challenging tasks to be accomplished. The real-time simulation of the blood flow and its interaction with surgical devices will not only achieve higher fidelity for the simulator, but also provide a promising way to perform hydrodynamics analysis for procedures like aneurysm embolization. Computational modelling of the blood vessels (42, 43) can enable us to understand the blood dynamics while the guidewire is travelling through the vessel, which helps in the research of cardiovascular condition during treatment. However, most of the existing methods (e.g., computational fluid dynamics) suffer from performance issues and cannot be used for interactive simulation. Our future work will focus on finding solutions to achieve both numerical accuracy and real-time efficiency for blood flow simulation.

## Data availability statement

The original contributions presented in this study are included in the article/Supplementary material, further inquiries can be directed to the corresponding author.

## Ethics statement

Ethical review and approval was not required for this study in accordance with the local legislation and institutional requirements.

## Author contributions

HW designed and conducted the experiments, analyzed the results, and wrote the manuscript. JW raised critical comments and edited the manuscript. Both authors contributed to the article approved the submitted version.
